# Prevalence, Awareness, Treatment and Control of Diabetes Mellitus—A Population Based Study in Shanghai, China

**DOI:** 10.3390/ijerph13050512

**Published:** 2016-05-19

**Authors:** Yuchen Qin, Rui Wang, Xiuqiang Ma, Yanfang Zhao, Jian Lu, Cheng Wu, Jia He

**Affiliations:** Department of Health Statistics, Second Military Medical University, No. 800 Xiang Yin Road, Shanghai 200433, China; qinyc10@163.com (Y.Q.); wangxusheng1948@163.com (R.W.); horse_ma88@yeah.net (X.M.); 15921636731@163.com (Y.Z.); 13564669464@163.com (J.L.); wucheng_wu@126.com (C.W.)

**Keywords:** diabetes, prediabetes, prevalence, awareness, control, HbAlc

## Abstract

In this study, we aimed to investigate the prevalence, awareness, treatment, and control of diabetes in Shanghai, China. A sample of 3600 residents aged from 18 to 80 years selected by a randomized stratified multiple-stage sampling method in Shanghai was investigated, with blood samples collected. Diabetes was defined as fasting plasma glucose (FPG) ≥ 7.0 mmol/L, or glycated haemoglobin (HbA1c) ≥ 6.5% (48 mmol/mol), or previous diagnosis by a physician. Adequate control of diabetes was taken as a level of HbA1c < 7.0% (53 mmol/mol) among people with treated diabetes. Multivariable regression analysis was used to explore associated factors for diabetes and prediabetes. In the 3136 participants suitable for analysis, the prevalences of diabetes, prediabetes, and previously diagnosed diabetes were 15.91%, 37.37%, and 4.46%, respectively. Among those with diabetes, only 28.06% were aware of their condition, 25.85% were currently undergoing medication treatment, and 12.42% achieved glycaemic control. Logistic regression showed that old age, preobesity, obesity, elevated triglyceride (TG), elevated C-reactive protein (CRP), and lower education level were associated with an increased risk of diabetes; old age, obesity, elevated TG, and elevated low-density lipoprotein (LDL) were associated with an increased risk of prediabetes, while male sex and rural residence were associated with a decreased risk of prediabetes. In summary, the state of diabetes in China is alarming; the rates of awareness, treatment, and control were relatively low. More efforts should be made to promote the prevention and control of diabetes in china.

## 1. Introduction

Diabetes has become a major public health problem in China [1,2]. A cross-sectional survey of 213,515 residents aged 25–64 years in 19 provinces and areas of China in 1994 found that the prevalence of diabetes in China was 2.5% [3]. Compared with developed countries, the prevalence of diabetes in China was quite low; however, with economic development and modernized lifestyle, it increased evidently. The prevalence in 1994 was three times what it was in 1980 (0.7%) [4]. The data from adults aged 20 years or older who participated in the China National Nutrition and Health Survey, 2002 (*n* = 47,729) showed that the prevalence of diabetes in Chinese adults was 2.7% [5]. In the national survey of 46,239 adults aged 20 years or older from June 2007 to May 2008, the age-standardized prevalence of diabetes was 9.7% [6], more than three times that in 2002. And the most recent national survey in 2010 reported that the overall prevalence of diabetes was estimated to be 11.6% [7]. Nevertheless, in these surveys, the screening methods and definitions of diabetes were different; the surveys before 1997 used fasting plasma glucose (FPG) cutoff for diabetes diagnosis of 7.8 mmol/L instead of 7.0 mmol/L, which might lead to an underestimate of diabetes; the survey in 2002 used fasting glucose as a screening test for diabetes, the survey in 2007 used oral glucose-tolerance test (OGTT) for screening [3,4,5,6], and the survey in 2010 diagnosed diabetes according to the American Diabetes Association(ADA) 2010 criteria [7]. Although these outcomes might not be compared directly, these data definitely indicated a rapid increase in diabetes as well as the increasing huge burden in the Chinese population. Shanghai, as an international metropolis, is a typical representative of the most developed area in China. The residents of Shanghai tend to have a relatively higher income, higher education level, and can easily get better health services. However, they also experience many risk factors, such as unhealthy eating habits, high stress, sedentary behaviors, *etc*. Although there have been a number of studies investigating the prevalence of diabetes and prediabetes in urban and rural areas in China [6,7,8,9] (Table 1), many of these did not provide information about the awareness, treatment, and control of diabetes. In addition, a representative survey that specifically targets developed areas is lacking. The aim of the current study was to estimate the prevalence of awareness, treatment, and control of diabetes mellitus based on fasting glucose and glycated haemoglobin (HbA1c) in Shanghai, China.

## 2. Materials and Methods

### 2.1. Study Design and Sample

The study was part of a recent population-based epidemiology survey. The details of the design were published by Yan *et al.* [16], and were briefly described here. We used a randomized stratified multiple-stage sampling method to select a representative sample of adults in Shanghai. Because approximately half of the residents lived in rural areas (54.2%) [16], at the first sampling stage, one district in urban areas (Hongkou District) and one district in rural stratum (Baoshan District) were randomly selected. Then following the sequence of block/town—residential area/village, 13 residential areas from two blocks in Hongkou District and four villages from two towns in Baoshan District were sampled, a total of 3600 residents aged from 18 to 80 years were sampled from these residential areas/villages, stratified by age and sex according to the data of the Fifth Population Census in Shanghai, 2000 [17]. In fact, 3153 participants completed the survey, with a response rate of 87.58%. Seventeen participants were deleted from the analysis because of missing fasting plasma glucose and HbA1c records. At last, 3136 participants were included in the analysis.

### 2.2. Data Collection

The survey was conducted from April 2007 to January 2008. The sampled participants were gathered to the local hospitals to conduct the investigation. The study was approved by the Second Military Medical University Ethics Committee (No. 20070011). All participants signed a written informed consent before participation.

After at least 10 h overnight fasting, venous blood samples were collected from each participant and analyzed in the central laboratory of Changhai hospital of Shanghai. FPG was measured by the glucose oxidase method (DiaSys Diagnostic Systems GmbH, Holzheim, Germany), total cholesterol (TC) by the cholesterol oxidase method (ProDia diagnostics Germany, Freiburg, Germany), triglyceride (TG) by the glycerophoshoric acid oxidase method (ProDia diagnostics Germany), HbA1c by the ion exchange liquid phase chromatography method (Bio-Rad Laboratories, Inc., Hercules, CA, USA), low-density lipoprotein cholesterol (LDL) and high-density lipoprotein cholesterol (HDL) by the enzymatic method (Daiichi Pure Chemical Co., Ltd., Tokyo, Japan). C-reactive protein (CRP) was measured with immunonephelometry on a special protein analyzer (Beckman Coulter, Inc., Brea, CA, USA).

A self-finished questionnaire was used to collect the demographic information, including resident region, gender, age, weight, height, and the diagnosis and treatment of diabetes, hypertension, and cardiovascular diseases. Body mass index (BMI) was calculated from height and weight and the WHO criterion for Asian populations was used [18], which are: (1) <18.5 kg/m^2^: underweight; (2) 18.5–22.9 kg/m^2^: normal weight; (3) 23–27.4 kg/m^2^: preobesity and (4) ≥27.5 kg/m^2^: obesity.

### 2.3. Disease Definition

Previously diagnosed diabetes was identified by the subjects’ positive response to the question, “Have you been diagnosed by a physician with diabetes?” Diabetes was defined as FPG ≥ 7.0 mmol/L, HbA1c ≥ 6.5% (48 mmol/mol) [19], or previous diagnosis by a physician. Prediabetes was defined as 5.6 mmol/L≤ FPG < 7.0 mmol/L, or 5.7% (39 mmol/mol) ≤ HbAlc < 6.5% (48 mmol/mol) [19]. The normal participants were defined as FPG < 5.6 mmol/L, HbA1c < 5.7% (39 mmol/mol), and no previously diagnosed diabetes. Awareness of diabetes was defined as self-reported previous physician-diagnosed diabetes. Treatment of diabetes was defined as self-reported use of antidiabetic medications. Adequate control of diabetes was taken as a level of HbA1c < 7.0% (53 mmol/mol) among people with treated diabetes [7].

### 2.4. Statistical Analysis

All questionnaires were doubly input into the database by two independent professional data processors using software EpiData 3.1. Statistical Analysis System (SAS) 9.3 (SAS Institute, Cary, NC, USA) was used for analyzing the data. All hypothesis tests used two-side tests and a *p*-value less than 0.05 was considered to be statistically significant. Pearson’s χ^2^ test was used for categorical variables, and the Cochra–Mantel–Haenszel test for ranked variables. Continuous variables among the diabetes group, the prediabetes group, and the normal group were analyzed by analysis of variance (ANOVA) with Student–Newman–Keuls test (SNK-q) for multiple comparisons, or Kruskal–Wallis test with Bonferroni for multiple comparisons, according to whether normality and homogeneous variance assumptions were met or not. Logistic regression was used to analyze the associated factors of diabetes.

## 3. Results

### 3.1. Characteristics of Participants

Among the 3136 participants with a mean age of 48 years, 1743 (55.58%) were female and 1393 (44.42%) were male. Approximately half of the participants lived in rural areas. The prevalence of obesity, preobesity, and underweight was 10.41%, 40.23%, and 6.19%, respectively; only 1352 (43.17%) had normal BMI (Table 2).

### 3.2. Prevalences of Diabetes and Prediabetes

The overall prevalences of diabetes and prediabetes were 15.91% (95% CI: 14.63%–17.19%) and 37.37% (95% CI: 35.68%–39.07%), respectively. However, if HbA1c was not considered, the prevalences of diabetes and prediabetes fell down to 7.84% (95% CI: 6.90%–8.79%) and 15.43% (95% CI: 14.17%–16.70%), respectively. The prevalences of previously diagnosed diabetes, FPG ≥ 7.0 mmol/L and HbA1c ≥ 6.5% (48 mmol/mol) were 4.46%, 6.09% and 13.14%, respectively (Table 2).

The prevalence of diabetes was similar between males (16.87%) and females (15.15%) (*p* = 0.19), and the prevalence of prediabetes was higher in females (40.62%) than in males (33.31%) (*p* < 0.001). However, if HbA1c was not considered, the males had a higher prevalence of diabetes than the females (9.48% *vs*. 6.54%, *p* = 0.002). Among the 3136 participants, 3.79% of women and 5.31% of men had been previously diagnosed with diabetes; 5.05% of women and 7.39% of men had FPG at or above 7.0 mmol/L; 12.34% of women and 14.14% of men had HbA1c at or above 6.5% (48 mmol/mol).

**Figure 1:**
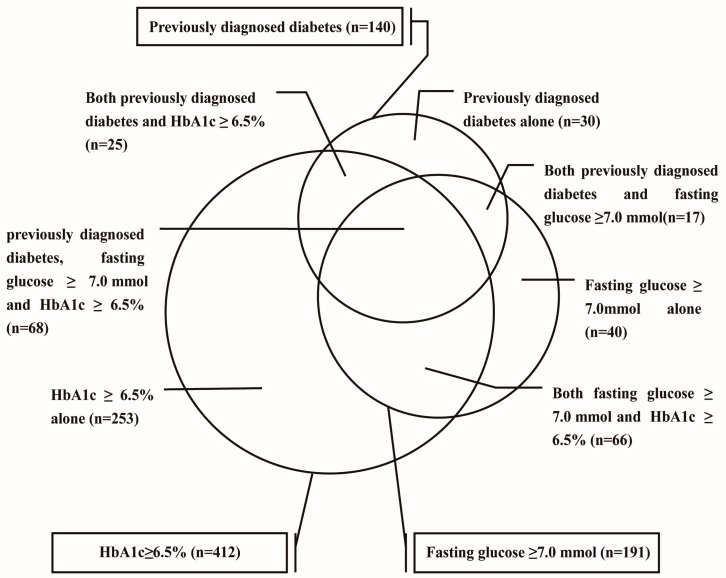
Overlap among participants with previously diagnosed diabetes, participants with HbA1c ≥ 6.5% (48 mmol/mol) and participants with fasting glucose ≥7.0 mmol.

In the 140 (28.06%) participants with previously diagnosed diabetes, 93 (66.43%) had HbA1c at or above 6.5% (48 mmol/mol), 85 (60.28%) had FPG no less than 7.0 mmol/L, and only 30 (21.43%) had normal HbA1c and FPG. In the 191 participants with FPG at or above 7.0 mmol/L, 85 (44.50%) had been diagnosed with diabetes previously, 134 (70.16%) had HbA1c no less than 6.5% (48 mmol/mol). Among the 412 participants with HbA1c at or above 6.5% (48 mmol/mol), 134 (32.52%) had FPG no less than 7.0 mmol/L, 93 (22.57%) had been diagnosed with diabetes (Figure 1). In the 359 newly diagnosed diabetes patients, only 66 patients (18.38%) were diagnosed by both FPG and HbA1c, 253 (70.47%) were diagnosed by HbA1c alone, and 40 (11.14%) were diagnosed by FPG alone.

With increasing age, the prevalence of diabetes increased from 6.79% (18–39 years), 13.97% (40–59 years) to 29.21% (60–80 years) in females, and 7.51% (18–39 years), 16.73% (40–59 years), to 29.47% (60–80 years) in males. No significant differences were found for the prevalences of diabetes between males and females in the 18–39 (*p* = 0.69), 40–59 (*p* = 0.12) and 60–80 (*p* = 0.94) years age groups. The same pattern was not found among those with prediabetes. In females, the prevalence of prediabetes in the 40–59 years age group was the highest (45.20%), followed by the 60–80 years group (37.64%), and the lowest was seen in the 18–39 years age group (33.97%); in males, the prevalence increased with increasing age, from 24.40% (18–39 years), 36.33% (40–59 years), to 37.19% (60–80 years). The females had a higher prevalence of prediabetes than males in the 18–39 (*p* = 0.003) and 40–59 (*p* < 0.001) years age groups, but not in the 60–80 years age group (*p* = 0.91).

Both diabetes and prediabetes have been found to be more prevalent in urban than in rural populations (*p* = 0.01 and *p* < 0.001, respectively). The prevalence of diabetes in urban regions for people aged 18–39 years old was higher than in rural areas (9.01% *vs.* 4.88%, *p* = 0.02), but for people 60–80 years old, the prevalence of diabetes in the urban region was much lower (21.86% *vs.* 36.36%, *p* < 0.001). The prevalence of diabetes increased with increasing BMI (*p* < 0.001), but there was no difference in the prevalence of prediabetes among different BMI groups (*p* = 0.31).

### 3.3. Awareness, Treatment, and Control of Diabetes

**Figure 2:**
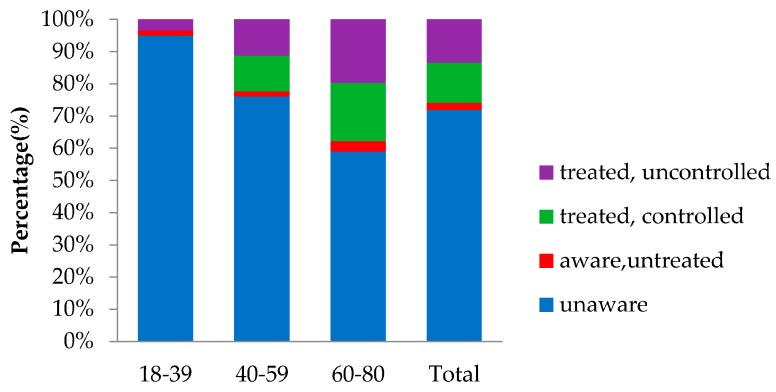
Age- and Sex-Standardized Rates of Awareness, Treatment, and Control of diabetes mellitus.

**Figure 3:**
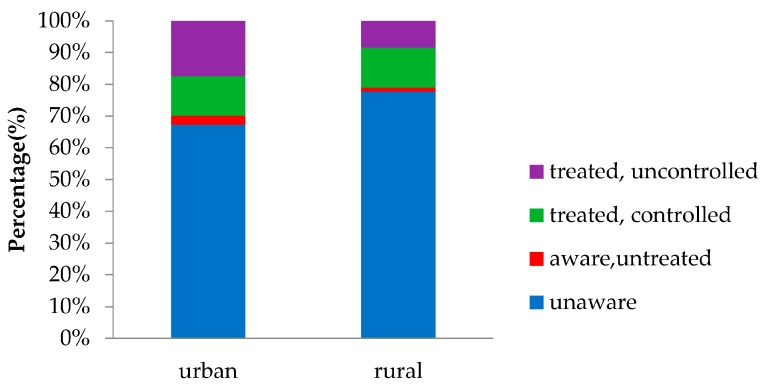
Rates of Awareness, Treatment, and Control of diabetes mellitus in urban and rural areas.

As shown in Figure 2, in the 499 participants with diabetes, only 140 (28.06%) were aware of their condition, leaving 359 (71.94%) previously unknown. As for residents in the 18–39 age group, the rate of awareness was extremely low (5.00%). Grouped by region, residents living in rural areas were more likely to be unware of their diabetes condition than in urban areas (77.68% *vs.* 67.27%, *p* = 0.01) (Figure 3). Among the patients with previously diagnosed diabetes, 11 (7.86%) had no treatment, 119 (85%) take oral anti-diabetic agents, 14 (10%) were treated with insulin, 38 (27.14%) were treated with diet therapy, and four (2.86%) used traditional medicine. Eighty (57.14%) patients were treated only with oral anti-diabetic agents, six (4.29%) were treated only with insulin, and the other patients were treated with multiple treatments. Among those treated, 62 (48.06%) had their HbA1c controlled to a concentration of less than 7.0% (53 mmol/mol). As for residents with diabetes, the proportion of people living in urban areas who could control their diabetes was much higher than people living in rural areas (59.57% *vs.* 41.46%, *p* = 0.048) (Figure 3).

### 3.4. Associated Factors of Prediabetes and Diabetes

Compared with normal participants, the participants with diabetes and prediabetes were older and with higher BMI. It was shown that TC, TG, LDL, and CRP were higher in the diabetes group than those in the normal group, with HDL lower in the diabetes group (*p* < 0.001). The participants with prediabetes had higher TC, TG, LDL, and CRP than the normal participants (*p* < 0.001) (Table 3).

After further analysis by logistic regression (Table 4), it is reported that ages 40–59 years (OR = 2.07; 95% CI: 1.50–2.86; *p* < 0.001), ages 60–80 years (OR = 4.73; 95% CI: 3.26–6.87; *p* < 0.001), preobesity (OR = 1.28; 95% CI: 1.01–1.62; *p* = 0.04), obesity (OR = 2.34; 95% CI: 1.71–3.21; *p* < 0.001), elevated TG (OR = 1.16; 95% CI: 1.07–1.26; *p* < 0.001), and elevated CRP (OR = 1.05; 95% CI: 1.02–1.07; *p* < 0.001) were significantly associated with an increased risk of diabetes. However, higher education (OR = 0.60; 95% CI: 0.37–0.97; *p* = 0.04) was significantly associated with a decreased risk of diabetes.

In addition, ages 40–59 years (OR = 1.96; 95% CI: 1.59–2.42; *p* < 0.001), ages 60-80 years (OR = 2.30; 95% CI: 1.71–3.09; *p* < 0.001), obesity (OR = 1.79; 95% CI: 1.32–2.43; *p* < 0.001), elevated TG (OR = 1.13; 95% CI: 1.04–1.24; *p* = 0.01), and elevated LDL (OR = 1.25; 95% CI: 1.06–1.48; *p* = 0.01) were significantly associated with an increased risk of prediabetes, while male sex (OR = 0.67; 95% CI: 0.52–0.86; *p* = 0.0019) and rural residence (OR = 0.60; 95% CI: 0.50–0.72; *p* < 0.001) were significantly associated with a decreased risk of prediabetes.

## 4. Discussion

Our results indicated that the prevalence of diabetes in Shanghai, representing the most developed area in China, which was defined as FPG ≥ 7.0 mmol/L, HbA1c ≥ 6.5% (48 mmol/mol), or previous diagnosis by a physician, was quite high: 15.91%. Moreover, it was 7.84% even without the consideration of HbA1c. The prevalence of prediabetes was also quite high, 37.37% if both FPG and HbA1c were considered. The results were higher than the China National Diabetes and Metabolic Disorders Study from 2007 to 2008, which showed that the prevalence of diabetes and prediabetes were 9.7% and 15.5%, respectively [6]. Compared with the newest nationally-representative cross-sectional study in 2010, which indicated the national average prevalence of diabetes and prediabetes were 11.6% and 50.1%, respectively, Shanghai had a higher prevalence of diabetes and a lower prevalence of prediabetes. Two reasons can probably explain these differences: firstly, our study did not use the OGTT, which could lead to the underestimation of diabetes and prediabetes. Secondly, according to previous studies, the prevalence of diabetes was higher in economically-developed regions than in the intermediately-developed and underdeveloped regions, but the prevalence of prediabetes was negatively correlated with socioeconomic level [6,7]. So Shanghai, as a representative of an economically-advanced region of China, which is generally a developing country, could display a relatively higher prevalence of diabetes and lower prevalence of prediabetes.

In our study population, only 140 participants (28.06%) knew that they had diabetes, leaving 71.94% undiagnosed. The outcomes were higher than the national average in 2001 (23.66% aware) and slightly lower than the national average in 2010 (30.10% aware). In addition, the awareness rate for residents under 40 years old was extremely low (5.00%). Compared with America, whose rate of awareness was 71% in 2002 [9] and 72% in 2014 [20], there was still a long way for Shanghai to cover in diabetes prevention and control, even if the socioeconomic condition of Shanghai was already at a relatively high level. Moreover, only approximately half of patients (48.60%) treated for diabetes had adequate glycemic control. Although the result is higher than the national average control rate in 2010 (38.6%), there was still much room for improvement, especially for residents living in rural areas who had a relatively lower awareness rate and control rate compared with people living in urban areas. The differences in the prevalence, awareness rate, and control rate of diabetes between urban and rural residents would probably originate from differences in living conditions, diet, physical activity, medical condition, knowledge about diabetes, *etc.* [21]. The high socioeconomic status, westernized diet and lifestyle, excessive work load, sedentary behaviors, and decreased physical activity of urban residents might contribute to increased risk for diabetes in urban areas, whereas greater accessible medical resources, higher level of self-awareness for health maintenance, and more knowledge about diabetes might improve the rate of awareness and control among urban residents.

The prevalence of diabetes increased with increasing age and increasing BMI, and more people with diabetes lived in urban than in rural areas. Logistic regression also showed that age, obesity, and TG were positively correlated with the risk of diabetes, while the education level was negatively correlated with the risk of diabetes. The reason might be that people with higher education could have a higher level of self-awareness for health maintenance and more knowledge about diabetes, so they would deliberately take some measures to keep their health condition and reduce the risk of diabetes. These findings were similar to other studies [3,6,12,22,23,24]. With rapid economic development, urbanization, and other socio-economic drivers, Chinese lifestyle has changed remarkably over the last decade. Chinese consumers have dramatically increased their consumption of meat, other livestock products, and fruits and have decreased consumption of grain-based foods, and people engage in less physical activity and have adopted a sedentary lifestyle [21,25,26,27]; all these lead to rising rates of obesity and dyslipidemia, which might cause the increasing prevalence of diabetes.

Our study has several limitations. The FPG and HbAlc, but not OGTT were used to diagnose diabetes in our survey, which may lead to an underestimation of diabetes. The newest national cross-sectional survey of diabetes in China has found that the combination of FPG and HbA1c could identify the diabetes of the most diabetic individuals (approximately 85%) identified by any of the three tests (*i.e.*, FPG, OGTT, and HbA1c) [28]. However, these results have already adequately revealed that diabetes has become a major public health problem in the China; the prevention and control of diabetes need improvement, even in economically-advanced areas with much better medical and health service facilities and conditions. In addition, because of the lack of OGTT, we cannot assess the agreement rate between OGTT and HbAlc, and it is difficult to evaluate the utility of an HbA1c value of 6.5% (48 mmol/mol) as a diagnostic tool for diabetes in the Chinese population.

## 5. Conclusions

In conclusion, based on our study, about one in six Chinese adults living in developed regions has diabetes, and one in three has prediabetes. Compared with the UK, which is a developed country, where diabetes affects about 1 in 20 adults, the state of diabetes in China is alarming. More efforts should be made to promote the prevention and control of diabetes in China, such as appropriate policy and intervention measurements, effective education programs, and healthy lifestyle with balanced diet, increasing physical activity and maintaining a healthy weight.

## Figures and Tables

**Table 1:** Studies for diabetes in China and America.

Study	Study Period	Sample Size	Age Range	Screening Method	Diagnostic Criteria	Prevalence of Diabetes
Population-based cross-sectional study in 19 provinces and areas in China [3]	1994–1995	213,515	25–64	OGTT, 2hBG, SR	WHO-1985	2.5%
Nationally representative sample of the general population in China [10]	2000–2001	15,540	35–74	FPG, SR	ADA	5.35%
China National Nutrition and Health Survey [5]	2002	47,729	20+	FPG, SR	ADA-2009	2.7%
Cross-sectional whole-population health survey in Tianjin rural area [11]	2004	769,792	35+	FBG, SR	WHO-1999	9.5%
Cross-sectional study on rural residents in North China [12]	2005	1058	20+	OGTT	WHO-1999	7.09%
National Health and Nutrition Examination Survey in America [13]	2005–2006	2806	20+	OGTT	ADA-1997	12.9%
China National Diabetes and Metabolic Disorders Study [6]	2007–2008	46,239	20+	OGTT, SR	WHO-1999	9.7%
Cross-sectional study in Shanghai	2007–2008	3136	18–80	FPG, HbA1c, SR	ADA-2009	15.91%
Prevalence, awareness, treatment, and control of diabetes mellitus in rural China: results from Shandong Province [14]	2007	16,375	25+	FPG, OGTT	WHO-1999	3.5%
Guangdong Health Survey [15]	2010	3590	18+	FPG, HbA1c, OGTT	ADA-2009	21.7%
A cross-sectional survey in a nationally representative sample of 98,658 Chinese adults [7]	2010	98,658	18+	FPG, HbA1c, OGTT	ADA-2010	11.6%

OGTT, oral glucose tolerance test; FPG, fasting plasma glucose; FBG, fasting blood glucose; 2hBG, 2-h blood glucose; HbA1c: glycated haemoglobin; SR, self-report.

**Table 2:** Prevalences of diabetes and prediabetes (% (95% CI)).

Cohort	Previously Diagnosed and/or Fasting Plasma Glucose ≥ 7.0 mmol/L and/or HbA1c ≥ 6.5% (Diabetes, *n* = 499)	Previously Diagnosed Diabetes and/or Fasting Plasma Glucose ≥ 7.0 mmol/L (*n* = 246)	Previously Diagnosed Diabetes (*n* = 140)	Fasting Plasma Glucose ≥ 7.0 mmol/L (*n* = 191)	HbA1c ≥ 6.5% (*n* = 412)	Prediabetes (*n* = 1172)
Overall	15.91 (14.63, 17.19)	7.84 (6.9, 8.79)	4.46 (3.74, 5.19)	6.09 (5.25, 6.93)	13.14 (11.96, 14.32)	37.37 (35.68, 39.07)
Sex						
Female (*n* = 1743)	15.15 (13.46, 16.83)	6.54 (5.38, 7.7)	3.79 (2.89, 4.68)	5.05 (4.02, 6.08)	12.34 (10.79, 13.88)	40.62 (38.31, 42.93)
Male (*n* = 1393)	16.87 (14.9, 18.84)	9.48 (7.94, 11.01)	5.31 (4.13, 6.49)	7.39 (6.02, 8.77)	14.14 (12.31, 15.97)	33.31 (30.83, 35.78)
*p*-value	0.190	0.002	0.040	0.006	0.137	<0.001
Age (year)						
18–39 (*n* = 844)	7.11 (5.38, 8.84)	1.66 (0.8, 2.52)	0.36 (0, 0.76)	1.42 (0.62, 2.22)	6.40 (4.75, 8.05)	29.74 (26.66, 32.82)
40–59 (*n* = 1651)	15.20 (13.47, 16.93)	7.45 (6.18, 8.72)	3.63 (2.73, 4.54)	6.06 (4.91, 7.21)	12.48 (10.88, 14.07)	41.25 (38.87, 43.62)
60–80 (*n* = 641)	29.33 (25.8, 32.85)	17.00 (14.1, 19.91)	12.01 (9.5, 14.53)	12.32 (9.78, 14.87)	23.71 (20.42, 27.01)	37.44 (33.69, 41.19)
*p*-value	<0.001	<0.001	<0.001	<0.001	<0.001	<0.001
Region						
Urban (*n* = 1570)	17.52 (15.64, 19.4)	8.41 (7.03, 9.78)	5.73 (4.58, 6.88)	6.18 (4.99, 7.37)	15.16 (13.39, 16.93)	42.74 (40.29, 45.19)
Rural (*n* = 1566)	14.30 (12.57, 16.04)	7.28 (5.99, 8.57)	3.19 (2.32, 4.06)	6.00 (4.83, 7.18)	11.11 (9.55, 12.67)	31.99 (29.68, 34.3)
*p*-value	0.014	0.240	<0.001	0.837	<0.001	<0.001
BMI (kg/m^2^)						
<18.5 (*n* = 194)	10.31 (6.03, 14.59)	2.06 (0.06, 4.06)	1.55 (0, 3.28)	1.03 (0, 2.45)	9.28 (5.2, 13.36)	34.02 (27.35, 40.69)
18.5~ (*n* = 1352)	12.20 (10.46, 13.95)	5.03 (3.86, 6.19)	3.18 (2.25, 4.12)	3.92 (2.89, 4.95)	10.95 (9.28, 12.61)	36.46 (33.9, 39.03)
23~ (*n* = 1260)	17.54 (15.44, 19.64)	9.52 (7.9, 11.14)	5.48 (4.22, 6.73)	7.22 (5.79, 8.65)	13.81 (11.9, 15.71)	38.02 (35.34, 40.7)
≥27.5 (*n* = 326)	28.22 (23.34, 33.11)	16.56 (12.53, 20.6)	7.67 (4.78, 10.56)	13.80 (10.06, 17.55)	21.78 (17.3, 26.26)	41.10 (35.76, 46.45)
*p*-value	<0.001	<0.001	<0.001	<0.001	<0.001	0.313

**Table 3:** Characteristics of participants with prediabetes, diabetes, and normal participants.

Characteristic	Normal Participants (*n* = 1465)	Prediabetes (*n* = 1172)	Diabetes (*n* = 499)
Age			
Mean ± SD	44.53 ± 13.83	49.15 ± 13.39	54.09 ± 13.85
Median	45.00	48.00	53.00
Q1, Q3	35.00, 52.00	41.00, 57.00	45.00, 65.00
*p*-value	1	<0.001 *	<0.001 *
BMI			
Mean ± SD	22.74 ± 3.16	23.44 ± 3.31	24.19 ± 3.43
Median	22.58	23.27	24.06
Q1, Q3	20.42, 24.80	21.23, 25.39	21.88, 26.13
*p*-value	1	<0.001 *	<0.001 *
TC			
Mean ± SD	4.78 ± 1.42	5.03 ± 0.97	5.17 ± 1.16
Median	4.64	4.96	5.05
Q1, Q3	4.15, 5.26	4.34, 5.66	4.38, 5.73
*p*-value	1	<0.001 *	<0.001 *
TG			
Mean ± SD	1.31 ± 1.01	1.52 ± 1.19	1.80 ± 1.48
Median	1.06	1.20	1.46
Q1, Q3	0.75, 1.53	0.86, 1.77	0.98,2.17
*p-*value	1	<0.001 *	<0.001*
HDL			
Mean ± SD	1.40 ± 0.33	1.39 ± 0.33	1.33 ± 0.33
Median	1.35	1.34	1.27
Q1, Q3	1.15, 1.59	1.15, 1.58	1.09, 1.50
*p*-value	1	0.69 *	<0.001 *
LDL			
Mean ± SD	3.04 ± 0.80	3.27 ± 0.87	3.34 ± 0.88
Median	3.00	3.20	3.30
Q1, Q3	2.50, 3.50	2.65, 3.80	2.70, 3.90
*p*-value	1	<0.001 *	<0.001 *
GLU			
Mean ± SD	4.74 ± 0.47	5.27±0.73	6.95 ± 2.65
Median	4.70	5.30	6.20
Q1, Q3	4.40, 5.10	4.70, 5.80	5.30, 7.80
*p*-value	1	<0.001 *	<0.001 *
HbA1c			
Mean ± SD	5.28 ± 0.27	5.78 ± 0.32	7.98 ± 3.64
Median	5.30	5.80	7.00
Q1, Q3	5.10, 5.50	5.70, 6.00	6.50, 8.30
*p*-value	1	<0.001 *	<0.001 *
CRP			
Mean ± SD	1.17 ± 2.73	1.47±3.30	2.46 ± 7.03
Median	0.47	0.61	0.91
Q1, Q3	0.23, 1.13	0.31,1.46	0.38, 2.22
*p*-value	1	<0.001 *	<0.001 *

Q1, first quartile; Q3, third quartile; SD, standard deviation. * Compared with normal participants (fasting plasma glucose < 5.6 mmol/L, and HbA1c < 5.7% (39 mmol/mol) and no previously diagnosed diabetes) using Bonferroni method with adjusted α = 0.01667. BMI: Body mass index; TC: Total cholesterol; TG: Triglyceride; HDL: High-density lipoprotein; LDL: Low-density lipoprotein; GLU, glucose; CRP: C-reactive protein.

**Table 4:** Logistic regression analysis for the associated factors of patients with diabetes or prediabetes.

Independent Variables	Diabetes		*p*-Value	Prediabetes		*p*-Value
	OR	95% CI		OR	95% CI	
Sex						
Female	1	Ref		1	Ref	
Male	1.61	0.86–1.57	0.33	0.67	0.52–0.86	0.0019
Age (year)						
<40	1	Ref		1	Ref	
40–59	2.07	1.50–2.86	<0.001	1.96	1.59–2.42	<0.001
60–80	4.73	3.26–6.87	<0.001	2.30	1.71–3.09	<0.001
Region						
Urban	1	Ref		1	Ref	
Rural	1.04	0.77–1.41	0.11	0.60	0.50–0.72	<0.001
BMI (kg/m^2^)						
Low weight (<18.5)	1.04	0.62–1.72	0.90	0.93	0.66–1.30	0.65
Normal weight (18.5~)	1	Ref		1	Ref	
Preobesity (23~)	1.28	1.01–1.62	0.04	1.04	0.87–1.25	0.66
Obesity (≥27.5)	2.34	1.71–3.21	<0.001	1.79	1.32–2.43	<0.001
Education						
Primary education or below	1	Ref		1	Ref	
Secondary education	1.04	0.77–1.41	0.79	0.95	0.73–1.23	0.69
Higher education	0.60	0.37–0.97	0.04	1.12	0.77–1.61	0.56
LDL	1.06	0.91–1.24	0.46	1.25	1.06–1.48	0.01
TG	1.16	1.07–1.26	<0.001	1.13	1.04–1.24	0.01
CRP	1.05	1.02–1.07	<0.001	1.02	0.99–1.04	0.27

OR, odds ratio; CI, confidence interval; Ref, reference group.

## Abbreviations

The following abbreviations are used in this manuscript:

FPG	fasting plasma glucose
HbA1c	glycated haemoglobin
OGTT	oral glucose-tolerance test
BMI	Body mass index
ANOVA	analysis of variance
SNK-q	Student-Newman-Keuls-q test
TC	total cholesterol
TG	triglyceride
LDL	low-density lipoprotein
HDL	high-density lipoprotein
GLU	glucose
CRP	C-reactive protein
